# Anesthesia triggers drug delivery to experimental glioma in mice by hijacking caveolar transport

**DOI:** 10.1093/noajnl/vdab140

**Published:** 2021-09-20

**Authors:** Lena Spieth, Stefan A Berghoff, Sina K Stumpf, Jan Winchenbach, Thomas Michaelis, Takashi Watanabe, Nina Gerndt, Tim Düking, Sabine Hofer, Torben Ruhwedel, Ali H Shaib, Katrin Willig, Katharina Kronenberg, Uwe Karst, Jens Frahm, Jeong Seop Rhee, Susana Minguet, Wiebke Möbius, Niels Kruse, Christian von der Brelie, Peter Michels, Christine Stadelmann, Petra Hülper, Gesine Saher

**Affiliations:** 1 Max-Planck-Institute of Experimental Medicine, Department of Neurogenetics, Göttingen, Germany; 2 Max-Planck-Institut für biophysikalische Chemie, Biomedizinische NMR, Göttingen, Germany; 3 Max-Planck-Institute of Experimental Medicine, Electron Microscopy Core Unit, Göttingen, Germany; 4 Max-Planck-Institute of Experimental Medicine, Department of Molecular Neurobiology, Göttingen, Germany; 5 Max-Planck-Institute of Experimental Medicine, Group of Optical Nanoscopy in Neuroscience, Göttingen, Germany; 6 Westfälische Wilhelms-Universität Münster, Institute of Inorganic and Analytical Chemistry, Münster, Germany; 7 Albert-Ludwigs-University of Freiburg, Faculty of Biology, Freiburg, Germany. Signalling Research Centres BIOSS and CIBSS, Freiburg, Germany. Center of Chronic Immunodeficiency CCI, University Clinics and Medical Faculty, Freiburg, Germany; 8 University Medical Center, Center for Nanoscale Microscopy and Molecular Physiology of the Brain (CNMPB), Göttingen, Germany; 9 University Medical Center Göttingen, Institute for Neuropathology, Göttingen, Germany; 10 University Medical Center Göttingen, Institute for Neurosurgery, Göttingen, Germany; 11 University Medical Center Göttingen, Institute for Anesthesiology, Göttingen, Germany; 12 Klinikum Oldenburg, Oldenburg, University Hospital, Germany

**Keywords:** blood, brain barrier, chemotherapy, drug delivery, general anesthesia, glioblastoma

## Abstract

**Background:**

Pharmaceutical intervention in the CNS is hampered by the shielding function of the blood–brain barrier (BBB). To induce clinical anesthesia, general anesthetics such as isoflurane readily penetrate the BBB. Here, we investigated whether isoflurane can be utilized for therapeutic drug delivery.

**Methods:**

Barrier function in primary endothelial cells was evaluated by transepithelial/transendothelial electrical resistance, and nanoscale STED and SRRF microscopy. In mice, BBB permeability was quantified by extravasation of several fluorescent tracers. Mouse models including the GL261 glioma model were evaluated by MRI, immunohistochemistry, electron microscopy, western blot, and expression analysis.

**Results:**

Isoflurane enhances BBB permeability in a time- and concentration-dependent manner. We demonstrate that, mechanistically, isoflurane disturbs the organization of membrane lipid nanodomains and triggers caveolar transport in brain endothelial cells. BBB tightness re-establishes directly after termination of anesthesia, providing a defined window for drug delivery. In a therapeutic glioblastoma trial in mice, simultaneous exposure to isoflurane and cytotoxic agent improves efficacy of chemotherapy.

**Conclusions:**

Combination therapy, involving isoflurane-mediated BBB permeation with drug administration has far-reaching therapeutic implications for CNS malignancies.

**Key Points**

- Moderate and short-term isoflurane exposure safely and reversibly opens the BBB in mice.
- Isoflurane triggers caveolar transport across the BBB.
- In combination with cisplatin chemotherapy, isoflurane reduces growth of GL261 tumors.

**Importance of the Study**

The blood–brain barrier (BBB) prevents efficient delivery of many drugs into the brain. However, general anesthetics such as isoflurane penetrate the blood–brain barrier to induce clinical anesthesia. Here, we show that isoflurane triggers caveolar transport across brain endothelial cells facilitating brain drug import. When applied at an optimal dosage, BBB function re-established directly after switching off anesthesia, which is of high clinical relevance. In a treatment trial of glioblastoma in mice, the combination of cytotoxic and optimized isoflurane application improves tumor therapy, which reveals the translational significance of this study. We hypothesize that our findings have implications for the therapy of a broad range of neurological diseases.

Cerebrovascular endothelial cells restrict and control CNS import and export across the BBB.^1,2^ However, this shielding function of the BBB often compromises drug delivery to the CNS.^1^ Especially in glioblastoma, the combination of highly invasive tumor growth and insufficient delivery of cytotoxic agents causes the poor prognosis for this fatal disease.

Approaches to facilitate noninvasive drug delivery to the CNS have revealed the double-edged sword nature of impairing BBB integrity. While a minor increase in BBB permeability that barely affects brain homeostasis might suffice for therapeutic drug import,^3,4^ a profound breach of the BBB is associated with severe side effects due to the entry of neurotoxic substances.^2^ Further, natural conditions of chronic BBB opening, as in inflammatory CNS disorders such as multiple sclerosis, lead to edema and neuroinflammation and can exacerbate disease.^5^ BBB injury is also implicated in neuroinflammation and postoperative neurocognitive defects following peripheral surgery under general anesthesia with volatile ethers such as isoflurane.^2,6–8^ Thus, a minimally invasive means to transiently increase BBB permeability is desired for controlled drug delivery into the brain. Here, we investigate the potential of isoflurane administration regimens to enhance BBB permeability without affecting endothelial cell survival and to facilitate drug delivery to the brain.

## Materials and Methods

### Animals

All animal experiments were performed in compliance with the ARRIVE guidelines and animal policies of the Max Planck Institute of Experimental Medicine, and were approved by the German Federal State of Lower Saxony (Lower Saxony State Office for Consumer Protection and Food Safety). Details to the use of animal material are provided in the Supplementary Methods.

### Patients

Ethical approval was obtained from the ethics committee of the University Medical Center (#19/5/20). Informed consent was obtained from all patients. Details to the use of patient material are provided in the Supplementary Methods.

### Cell Culture

Primary mouse brain EC cultures were established from 7 days old mice or rats as described.^9^ Details to the use of cultured cells are provided in the Supplementary Methods.

### Super-resolution Microscopy

STED and SRRF super-resolution microscopy was applied as detailed in the Supplementary Methods.

### Histochemistry

Histochemical analyses were done as described^9^ and detailed in the Supplementary Methods.

### Protein Analysis

Tissue samples were processed according to standard procedures as detailed in the Supplementary Methods.

### Antibodies

The following antibodies were used: CD31/PECAM1 (Dianova Dia-310), AQP4 (Santa Cruz sc-20812), cFos (Santa Cruz sc-271243), tubulin (Sigma T7941), GFP (Rockland 600-101-215), CD3 (Serotec MCA1477), occludin (Thermo Fisher 710192), ZO-1 (Thermo Fisher 61-7300), and claudin-5 (Thermo Fisher 34-1600), actin (Sigma A3853), isolectin IB4 (Vectorlab DL-1207), Iba1 (Wako 019-19741), and arginase-1 (Santa Cruz sc-18351).

### Expression Analysis

Expression analyses were carried out as described^9^ and detailed in the Supplementary Methods. All primer sequences are listed in the Supplementary Table.

### Blood–brain Barrier Permeability

Measurements of BBB permeability were done as described^3,9^ and details are provided in the Supplementary Methods.

### Single Molecule Array

Neurofilament light chain (NFL) concentration in serum of mice or patients was determined using a Neurofilament-light Advantage assay kit (Quanterix) according to the manufacturer´s specification. In brief, serum samples were diluted 1:4 with sample diluent supplied with the kit, transferred to Quanterix microtiter plates, and analyzed by a SIMOA HD-1 Analyzer.

### Tumor Cell Implantation and Anti-tumor Treatment

Implantation of GL261 glioma cells and chemotherapy with cisplatin was done as detailed in the Supplementary Methods.

### MRI

To determine tumor size, MRI of mice with implanted glioma cells was done as detailed in the Supplementary Methods.

### Laser Ablation–Inductively Coupled Plasma—Mass Spectrometry (LA-ICP-MS)

To quantify cisplatin delivery to the brain, platinum was spatially localized and quantified by elemental bioimaging by means of laser ablation—inductively coupled plasma—mass spectrometry (LA-ICP-MS) on brain sections from mice treated with CP or CP plus isoflurane. Details are provided in the Supplementary Methods.

### Statistics

Statistical evaluation was done using GraphPad Prism (GraphPad Inc.), applying either unpaired Student’s *t* test for pairwise comparisons, ANOVA for comparisons of more than 2 groups or the Mann–Whitney test for nonparametric comparisons, as stated in the figure captions. ANOVA was combined with a Dunnet’s or a Tukey’s test to correct for multiple comparisons and evaluate individual groups. Data analysis was performed blinded to the experimental groups. All error bars show mean ± SEM or median ± interquartile range as indicated in the figure captions with individual values. *P* values are shown as *P < .05, **P < .01, ***P < .001.

## Results

### Isoflurane Interferes with the Lateral Organization of Lipids

**Figure 1.**
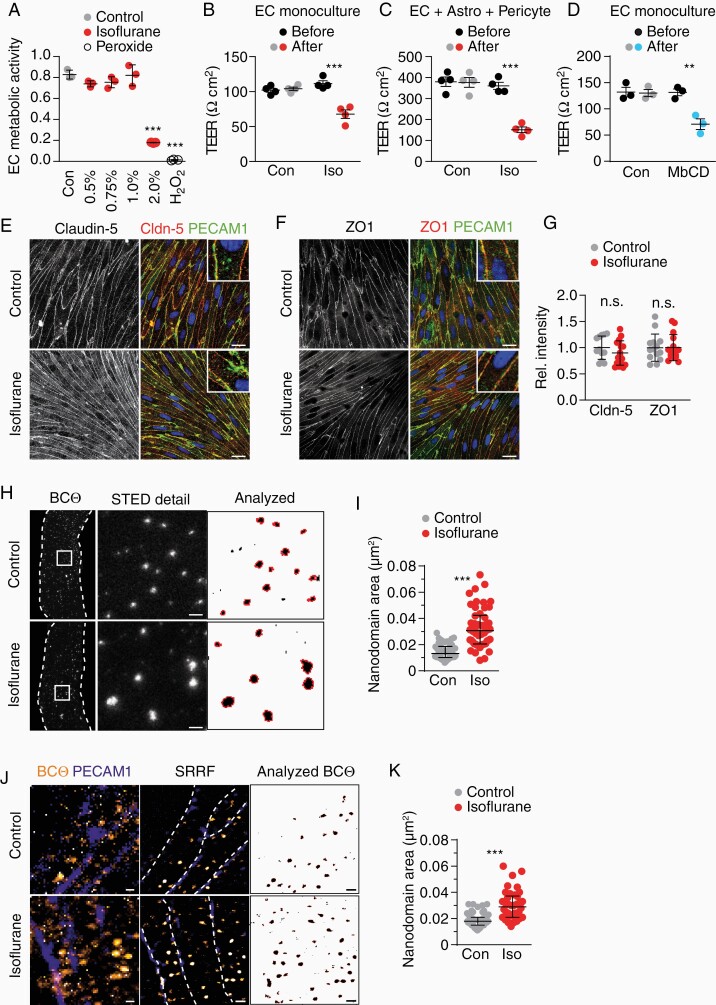
Isoflurane increases EC barrier permeability in vitro. (**A**) Mean viability ± SD of ECs treated with 0.5%–2% isoflurane (30 min) or peroxide as positive control (*n* = 3 experiments, 1-way ANOVA with Dunnett’s test), quantified by a WST1 assay that quantifies enzymatic activity of mitochondrial dehydrogenases present in viable cells. (**B**) Mean TEER ± SEM of EC cultures before and after 30 min 1% isoflurane treatment (*n* = 4 cultures, unpaired Student’s *t*-test). (**C**) Mean TEER ± SEM of triple cultures before and after 30 min 1% isoflurane treatment (*n* = 4 cultures, unpaired Student’s *t*-test). (**D**) Mean TEER ± SEM of EC cultures before and after 30 min 10 mM MbCD treatment (*n* = 3 cultures, unpaired Student’s *t*-test). (**E–G**) Co-immunofluorescence of PECAM1 with (**E**) claudin-5 and (**F**) ZO1 in EC cultures treated as in (B) (scale 20 µm) with (**G**) quantification of mean fluorescence intensity in *n* = 3 cultures. (**H**) STED images of ECs live-stained for cholesterol (BCtheta) after 30 min isoflurane treatment (cell borders, dashed line) with detailed area (scale 500 nm). Nanodomains included in the quantification are outlined in red (right). (**I**) Median size of BCtheta-positive nanodomains ± interquartile range (*n* = 50–51 cells from 3 experiments, Mann–Whitney test). (**J**) Confocal (left), chromatic aberration corrected SRRF (middle, cell borders, dashed line), and thresholded SRRF images (right) of endothelial cells treated as in (B) (scale 500 nm). (**K**) Median size of BCtheta-positive nanodomains ± interquartile range (*n* = 38–57 cells from 2 experiments, Mann–Whitney test).

Isoflurane exposure can impair endothelial cell (EC) survival, which in turn disrupts the BBB.^6^ However, it is unclear whether increased barrier permeability can occur independently of EC damage. When we treated primary ECs with increasing concentrations of isoflurane for 30 min, metabolic activity of ECs was unaffected up to 1% but decreased drastically at 2% isoflurane (**Figure 1A**). We then tested whether a sub-critical dose interfered with the barrier function of ECs by quantifying ionic conductance. Surprisingly, when measured directly after a 30-min treatment with 1% isoflurane, transendothelial electrical resistance (TEER) dropped to about 60% of pretreatment values, demonstrating that isoflurane acutely increases permeability of EC cultures, independent of EC viability (**Figure 1B**). Moreover, in an advanced barrier culture comprising EC, astrocyte, and pericytes, 1% isoflurane led to a similar decrease in TEER (**Figure 1C**), suggesting that the glial cells of the neurovascular unit in vitro did not counteract the isoflurane-mediated increase in barrier permeability.

In TEER measurements, the current can flow across the cells (transcellular path) and through the junctions between cells (paracellular path). Depleting cholesterol with methyl-β-cyclodextrin (MbCD) impairs tight junction integrity in an epithelial cell line.^10^ In our EC cultures, MbCD caused a drop in TEER values comparable to the isoflurane treatment (**Figure 1D**). Thus, we determined integrity of tight junction proteins in EC cultures treated with isoflurane as before, by immunolabeling after 24 h. However, location and abundance of the tight junction proteins claudin-5 and ZO1 in cell–cell junctions was comparable in isoflurane-treated and control cultures (**Figure 1E–G**), a finding that is in agreement with a study using an EC line.^11^ Although some gene transcripts related to EC tight junctions were downregulated in vitro (Supplementary Figure 1A) but not in vivo (see below), these findings suggest that isoflurane—in contrast to cyclodextrin—does not affect tight junction integrity and paracellular leakage.

Barrier leakiness could be mediated by increased transcellular passages that include micropinocytosis and clathrin-mediated as well as caveolin-mediated vesicular transport pathways.^12,13^ All these pathways depend on the integrity of plasma membrane lipids but only caveolae contain membrane lipid nanodomains, also termed lipid rafts, with their obligate component cholesterol.^14^ In model membranes, isoflurane interferes with the organization of lipid domains, which leads to disturbances in lipid mobility and increased membrane fluidity.^15,16^ We therefore hypothesized that in primary EC, isoflurane exposure could affect lipid nanodomains, which would result in enhanced transcytosis in our in vitro system. We stained primary ECs with BCtheta that binds cholesterol, predominantly when located in membrane lipid nanodomains.^4^ Using super-resolution stimulated emission depletion (STED) microscopy, applying a cut-off size of 3,600 nm^2^, the median area of BCtheta-labeled membrane lipid nanodomains in the plasma membrane of control EC cultures was 13,160 nm^2^. This corresponds to ~130 nm in diameter (**Figure 1H and I**, Supplementary Figure 1B and C), a size in accord with previous reports.^17^ Surprisingly, isoflurane (1%, 30 min) significantly increased the median area of these nanodomains to 30,670 nm^2^ (~198 nm in diameter), which is in the range of the maximum area in control-treated cells (29,010 nm^2^). In isoflurane-treated cells, the median fraction of the cell surface that was covered by BCtheta-labeled nanodomains, increased (Supplementary Figure 1D), suggesting that isoflurane exposure did not extract cholesterol from the membrane. Rather, this is compatible with dispersion of cholesterol molecules, analogous to decreasing the ratio of liquid-ordered to liquid-disordered domains in model membranes.^16^ By applying super-resolution radial fluctuations (SRRF), an analytical super-resolution microscopy technique that allows high-fidelity reconstructions, we confirmed the isoflurane-mediated increase in membrane lipid nanodomain size (**Figure 1J and K**, Supplementary Figure 2). These data suggest that our low-dose isoflurane treatment that did not interfere with EC viability, disturbed the organization of lipid nanodomains in plasma membranes of endothelial cells, which contributed to the increase in barrier permeability in vitro.

### Isoflurane Can be Utilized to Control BBB Permeability In Vivo

**Figure 2.**
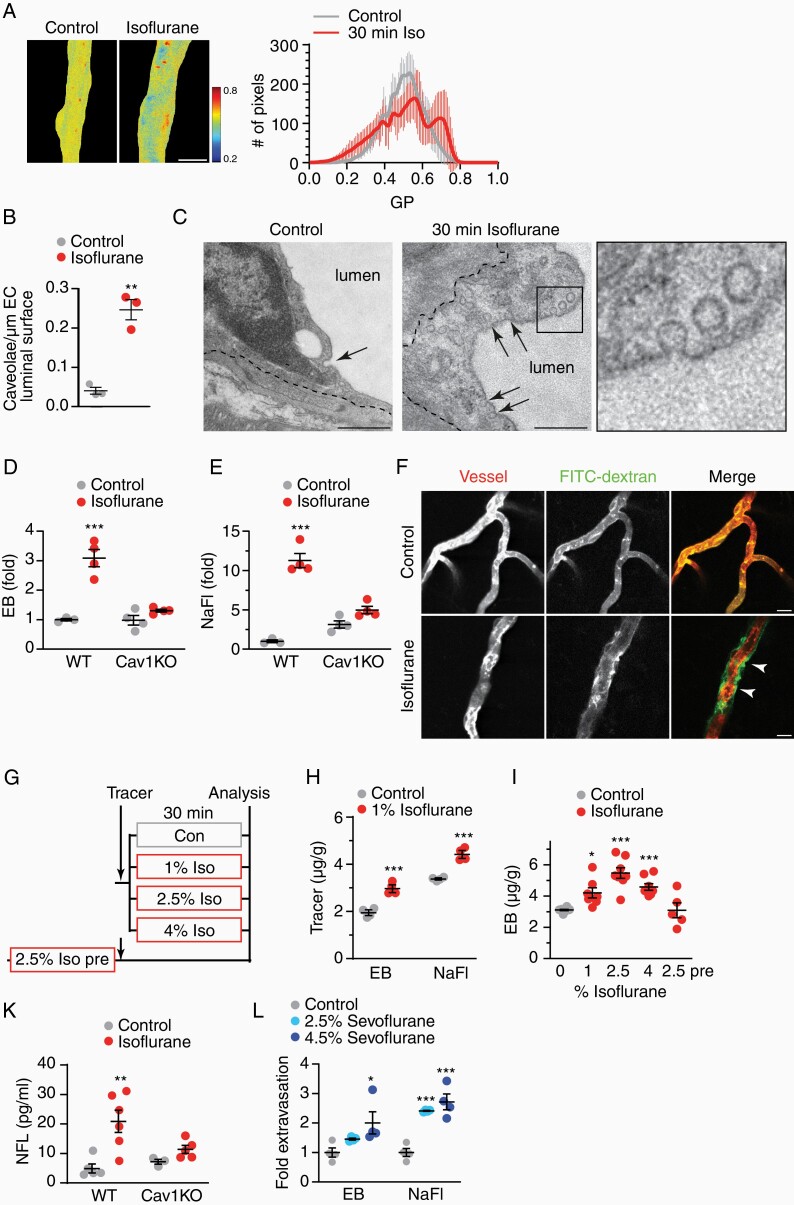
Isoflurane disturbs BBB tightness in vivo. (**A**) Membrane fluidity of capillaries in mice treated with isoflurane (2.5%, 30 min) visualized by GP of Laurdan shown in false color with mean GP ± SEM of *n* = 13 vessels per condition. (**B**) Mean density of luminal micro-invaginations ± SEM in mice exposed to isoflurane (39 and 41 capillaries from *n* = 3 animals, unpaired Student’s *t*-test). (**C**) Electron micrographs of caveolar profiles (arrows) in brain ECs (dashed line, abluminal EC membrane), given in detail in boxed area (scales 500 nm). (**D**) Mean fold extravasation of EB ± SEM in WT and caveolin-1 deficient (Cav1KO) mice treated with isoflurane (*n* = 3–4 animals, 2-way ANOVA with Tukey’s test). (**E**) Mean fold extravasation of NaFl ± SEM in WT and Cav1KO mice treated with isoflurane (*n* = 3–4 animals, 2-way ANOVA with Sidak’s test). (**F**) Representative images of FITC-dextran extravasation in mice exposed to isoflurane (arrowheads). DiI-mediated vessel paint stained capillaries (scale 25 µm). (**G**) Experimental paradigm of BBB permeability. Mice were exposed to increasing dosages of isoflurane directly after tracer injection. In the 2.5% Iso pre-paradigm, tracer was administered to mice following the isoflurane treatment. (**H**) Mean extravasation of tracers (µg/g brain weight) ± SEM (*n* = 4 animals, 1-way ANOVA with Dunnett’s test). (**I**) Mean extravasation of EB (µg/g brain weight) ± SEM (*n* = 5–10 animals, 1-way ANOVA with Dunnett’s test, indicated are significant changes to controls). (**K**) Mean serum NFL ± SEM in WT and Cav1KO mice treated with 2.5% isoflurane for 30 min (*n* = 3–6 animals, 2-way ANOVA with Sidak’s test).

Next, we asked whether isoflurane inhalation also alters the organization of membrane lipid domains of brain ECs in vivo. We treated mice with 2.5% isoflurane for 30 minutes and acutely stained the brain vasculature by Laurdan vessel paint during perfusion. The generalized polarization (GP) of Laurdan fluorescence determines membrane lipid-packing properties in bilayers and cell membranes, as the emission spectrum of this polarity-sensitive dye depends on the local order of lipids.^18,19^ In vessels of control mice, GP values revealed a largely uniform distribution (**Figure 2A**). This is expected, because the resolution limits of confocal microscopy do not allow visualization of membrane lipid nanodomains. In contrast, brain capillaries of isoflurane-treated mice showed an inhomogeneous GP distribution, pointing to local variances in membrane lipid order. This is compatible with our in vitro findings and with isoflurane-mediated perturbation of model membranes.^15,16^

We then investigated whether the disturbance of endothelial membrane lipids affected transcytosis pathways in vivo, focusing on caveolar transport. Caveolae are especially scarce in brain capillaries, contributing to the physiological BBB tightness.^20^ Caveolae formation is suppressed by the unique lipid composition in brain EC. Injury or interference with this lipid composition can disinhibit caveolar transcytosis and increase BBB permeability.^20–22^ To test the hypothesis that isoflurane facilitates caveolae formation, we used electron microscopy to quantify luminal flask-like micro-invaginations lacking a coat, which is typical for caveolae. As expected, in capillaries from control animals we found only few caveolar profiles on electron micrographs (Figure 2B and C). In contrast, in mice treated for 30 min with 2.5% isoflurane, the mean density of caveolar profiles increased ~4-fold. In extreme cases, EC showed an almost 30-fold increased density of transcytotic vesicles (Figure 2C), reminiscent of arteriolar EC.^21^ Notably, in both treatment groups, we only occasionally observed profiles consistent with clathrin-coated pits or with large macropinosomes, thus suggesting that isoflurane interfered with caveolar transport.

We then investigated whether the isoflurane-induced formation of caveolae caused BBB impairment. For this, we treated WT and caveolin-1-deficient mice (Cav1KO) that lack the principal component of caveolae, with isoflurane and determined BBB permeability by tracer molecule extravasation using a biochemical assay. We applied the small tracer sodium fluorescein (NaFl, 0.376 kDa) and the large tracer Evans blue (EB, bound to albumin, 67 kDa). EB is used as a specific marker for transcellular transport.^23^ Isoflurane-treated WT mice showed a robust 3-fold extravasation of EB and a more than 10-fold extravasation of NaFl, demonstrating that isoflurane indeed increased BBB permeability also in vivo (**Figure 2D and E**). Strikingly, caveolin-1-deficient mice were resistant to this isoflurane-mediated rise of BBB permeability (**Figure 2D and E**), pointing to caveolae as the molecular target of isoflurane treatment.

Caveolar transport can facilitate nonspecific passage of blood-borne molecules of a broad size range into the brain parenchyma. When we i.v. injected fluorescein isothiocyanate coupled to 70 kDa dextran (FITC-dextran), this large tracer extravasated to the perivascular space only in isoflurane-treated mice but not in controls (**Figure 2F**). We then analyzed tracer extravasation in response to increasing concentrations of isoflurane using our biochemical assay. Strikingly, significant extravasation of both tracers was evident at 1% isoflurane exposure, corresponding to a minimum alveolar concentration (MAC) of ~0.7 in adult mice (**Figure 2G and H**). The degree of extravasation correlated with isoflurane concentrations (**Figure 2I**, Supplementary Figure 3A), demonstrating that isoflurane exposure increased BBB permeability in a dose-dependent manner. Strongest extravasation occurred at 2.5% isoflurane (~1.8 MAC). NaFl extravasation even occurred when isoflurane was applied with a protocol that does not induce deep anesthesia in mice (Supplementary Figure 3B). It is known that isoflurane is rapidly cleared from the brain^24^ but it could potentially induce permanent damage at endothelial junctions. To test whether isoflurane exposure triggers a persistent increase in BBB permeability, we injected the tracer EB directly after termination of the isoflurane exposure (**Figure 2G**, 2.5% Iso pre). Surprisingly, in this paradigm tracer extravasation were at control levels (**Figure 2I**), which implies that the BBB resealed immediately after isoflurane withdrawal.

Adsorptive mediated transcytosis by caveolae usually transports positively charged cargo from the blood into the brain, with unknown transport activity in the opposite direction.^25^ To test whether isoflurane inhalation facilitated bidirectional passage, we monitored the concentration of neurofilament light chain (NFL) in serum of mice after isoflurane exposure by single molecule array analysis. While control mice showed, as expected, low steady-state NFL levels, serum NFL increased 4-fold in isoflurane-treated WT mice (**Figure 2K**). In contrast, isoflurane exposure did not increase serum NFL levels in caveolin-1-deficient mice, suggesting that the NFL export occurred via caveolae. Together, these findings demonstrate that an increase in BBB permeability induced by volatile anesthetics such as isoflurane can be monitored using the biomarker NFL, a clinically available diagnostic assay using serum samples.

We next tested serum NFL levels in human patients, who lacked signs of BBB impairment, before and after 1h exposure to inhalation anesthesia. We applied sevoflurane, which is related to isoflurane but is clinically more relevant at a routinely applied concentration of 0.8-1 MAC. However, a comparable general serum NFL increase as in mice was not observed (Supplementary Figure 3C). To examine whether differences between the applied anesthetics (isoflurane vs sevoflurane) played a role,^6^ we treated mice with sevoflurane, applying 2.5% and 4.5% relating to 1 MAC and 2.5 MAC in mice, and measured BBB permeability. Fold extravasation of the tracers Evans Blue and sodium fluorescein (NaFl) was comparable to isoflurane at the corresponding concentrations (**Figure 2L**), showing robust extravasation at the high dose and marginal extravasation at the low dose of sevoflurane. These findings demonstrate that both inhalation anesthetics can impair the BBB in mice. In addition to species differences, it is likely that the low dose in humans compared to mice (0.8-1 MAC vs 1.8 MAC) accounted for the observed discrepancy between patients and mice.

Together, these findings suggest that isoflurane exposure triggers the formation of caveolae that mediate transcellular transport across the BBB, involving import and export processes. When applied at clinically relevant concentrations, isoflurane in mice can be used as a tool to modulate BBB permeability.

### Prolonged Exposure to Isoflurane Induces Brain Edema

**Figure 3.**
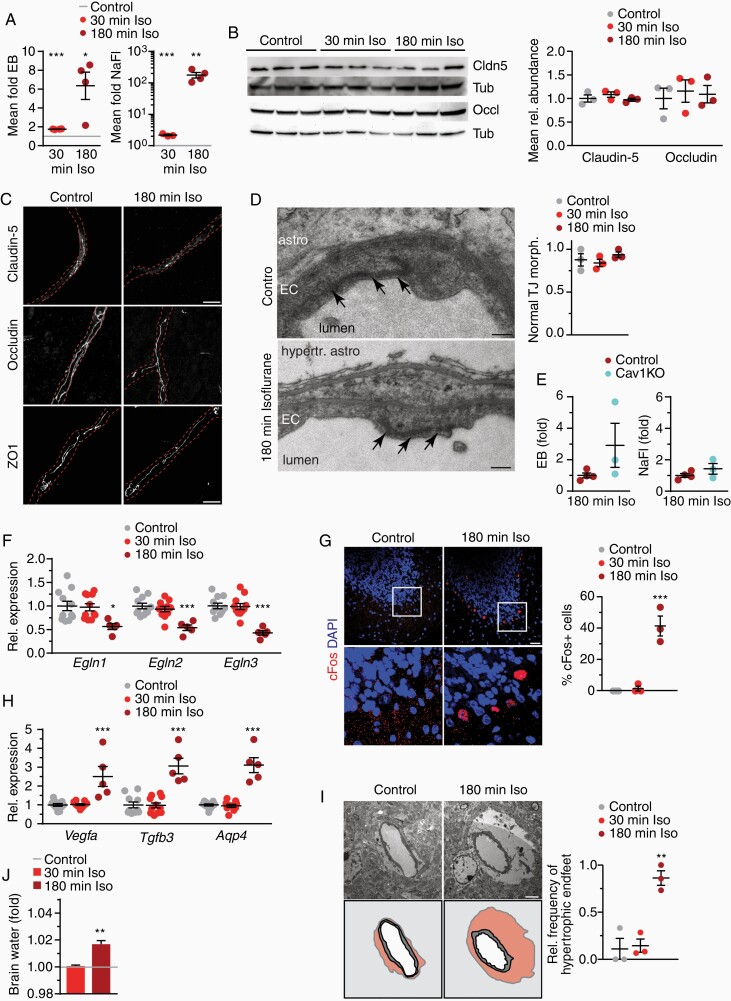
Prolonged exposure to isoflurane induces brain edema. (**A**) Extravasation of tracers ± SEM after 2.5% isoflurane compared to controls (gray line; *n* = 4 animals, 1-way ANOVA with Dunnett’s test). (**B**) Immunoblot of brain lysates from isoflurane-treated mice and for claudin-5, occluding, and tubulin with quantification (*n* = 3 animals). (**C**) Images of brain sections immunostained for tight junction proteins with vessel outline (dashed lines) from lectin co-staining (scale 10 µm). (**D**) Normal ultrastructure of EC tight junctions (arrows) (astro, astrocyte; hypertr. astro, hypertrophic astrocyte; scale 200 nm) with quantification of the mean ratio of normal-appearing tight junction profiles below (23–37 vessels from *n* = 3 animals). (**E**) Extravasation of tracers ± SEM after 2.5% isoflurane for 180 min in WT and Cav1KO mice (*n* = 3–4 animals). (**F**) Mean gene expression of *Egln1-3* ± SEM in brain from isoflurane-treated mice compared to controls (*n* = 5–11 mice, 1-way ANOVA with Dunnett’s test). (**G**) cFos immunostaining of cerebellum from mice treated with isoflurane shows frequent staining in Purkinje neurons (scale 25 µm) with quantification (*n* = 3 animals, 1-way ANOVA with Dunnett’s test). (**H**) Mean expression of genes encoding Hif1αtarget genes as indicated ±SEM in brain tissue of mice treated with isoflurane compared to controls (*n* = 5–11 mice, 1-way ANOVA with Dunnett’s test). (**I**) Swollen astrocyte endfeet around capillaries after 180 min of isoflurane administration (n astrocyte nucleus, v vessel, pseudocolored in the cartoon below, scale 2 µm) with quantification of mean frequency compared to controls (*n* = 3 animals, 1-way ANOVA with Dunnett’s test). (**J**) Mean brain water content indicative of edema in controls (gray line) or mice exposed to isoflurane (*n* = 4–5 animals, unpaired Student’s *t*-test to control animals).

Next, we asked whether a longer duration of isoflurane inhalation that is nevertheless in the clinically relevant range, can further impair BBB tightness. For this, we exposed mice to 2.5% isoflurane for 180 min in comparison to control and to 30 min exposures as applied before. The tracer extravasation increased about 6.4-fold (EB) or even 180-fold (NaFl) in mice after 180 min of isoflurane exposure, compared to 1.8- and 2.2-fold increases in mice exposed to isoflurane for 30 min, respectively (**Figure 3A**). We then tested whether impairment of tight junctions accounted for this strong increase in BBB permeability. However, transcript levels of genes related to endothelial junctions did not decrease after 180 min administration with isoflurane (Supplementary Figure 3D and E). Protein abundances of claudin-5 and occludin remained unchanged (**Figure 3B**). Immunolabeling revealed a normal continuous pattern of tight junctions (**Figure 3C**), and the ultrastructure of EC-EC contact sites showed normal electron dense junctional areas (**Figure 3D**). Together, these findings suggest that even the strongly enhanced tracer extravasation after prolonged isoflurane exposure does not cause (permanent) disruption of endothelial tight junctions.

We then examined whether the enhanced caveolae formation observed after 30 min of isoflurane administration also persisted at 180 minutes’ treatment. However, the density of caveolar profiles after prolonged isoflurane administration was comparable to controls (Supplementary Figure 3F). The expression of EC proteins involved in caveolar transcytosis, caveolin-1 and Mfsd2a (major facilitator superfamily domain containing 2a), which control the formation of caveolae,^20,26^ was downregulated at 180 min treatment times (Supplementary Figure 3G). Moreover, tracer extravasation after 180 min isoflurane was comparable in caveolin-1-deficient and WT mice (**Figure 3E**). These data suggest that caveolar transcytosis did not account for the strongly enhanced BBB permeability after prolonged isoflurane treatment.

Blood oxygen saturation remains stable throughout isoflurane exposure.^24^ Nevertheless, in vitro long-term isoflurane exposure induces Hif1αsignaling.^27^ We therefore tested whether 180 min of isoflurane anesthesia initiated a Hif1αresponse in the brain. Quantification of direct Hif1αtranscription factor activity in tissue is prone to preparation artifacts. In cell lines, gene silencing of *Egln1-3*, the prolyl-hydroxylases that mediate Hif1αturnover under physiological conditions, enhances HIF-dependent transcription.^28^ The transcript levels of all 3 enzymes were downregulated to 50% of control levels after 180 min, but not after 30 min of isoflurane exposure (**Figure 3F**). About 40% of Purkinje neurons of the cerebellum expressed the immediate early gene and HIF target cFos after 180 min of isoflurane exposure but not after 30 min (**Figure 3G**), implying an acute response. Moreover, expression of Hif1α target genes VEGFα, TGFβ3 and the water channel aquaporin-4 were robustly increased after 180 min isoflurane exposure (**Figure 3H**). Activated Hif1α signaling therefore likely contributed to the development of vasogenic edema as evidenced by the enhanced incidence of hypertrophic astrocyte endfeet on electron micrographs (**Figure 3I**) and increased brain water content (**Figure 3J**). In contrast, pericytes and microglia appeared normal in morphology and abundance, and inflammatory responses were not evident (Supplementary Figure 3H–J). Together, these data demonstrate that 3 h exposure to 2.5% isoflurane induces Hif1α-dependent transcription and causes brain edema that likely amplifies BBB impairment. This condition might contribute to the cognitive deficits observed after isoflurane exposure.^29,30^

### Isoflurane Improves Chemotherapy of Glioma

**Figure 4.**
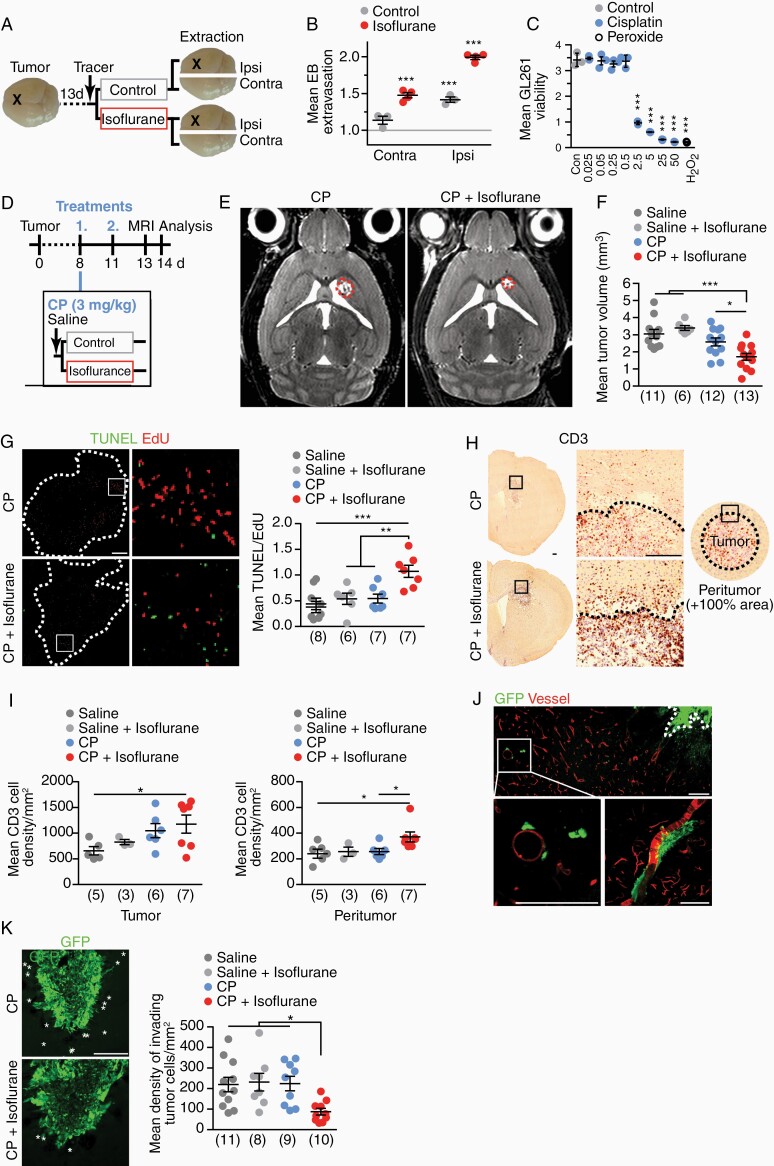
Isoflurane supports cisplatin treatment of glioma in mice. (**A**) At 14 d after GL261 tumor cells implantation (X), EB was i.v. injected and mice were treated with 2.5% isoflurane for 30 min. Extravasated tracer was quantified in the ipsilateral tumor-bearing or the contralateral hemispheres. (**B**) EB extravasation ± SEM in tumor-implanted mice treated as depicted in (A), normalized to control mice set to 1 (gray line; *n* = 3, 1-way ANOVA with Tukey’s test). (**C**) Viability of GL261 tumor cells ± SEM treated with increasing CP concentrations for 72 h measured (*n* = 3, 1-way ANOVA with Dunnett’s test). (**D**) Treatment scheme of glioma with CP chemotherapy with supportive isoflurane application. At 8 dpi and 11 dpi, mice received chemotherapy. Tumor volume was assessed by MRI at 13 dpi. Mice were euthanized at 14 dpi. (**E**) MRI images of mice treated as depicted in (D) with tumor outline as dashed line. (**F**) Tumor volume ± SEM (*n* = 6–13 as indicated in brackets, 1-way ANOVA with Dunnett’s test). (**G**) TUNEL and EdU colabeled brain section (tumor outline dashed, scale 200 µm) with boxed area enlarged on the right. TUNEL/EdU ratio ± SEM of tumors as readout of treatment efficacy (*n* = 6–8 as indicated in brackets, 1-way ANOVA with Dunnett’s test). (**H**) Tumor and peritumor areas used for quantification (scale 200 µm). (**I**) CD3+ T lymphocytes in tumor (left) and peritumor areas (right) ± SEM (*n* = 6–13 as indicated in brackets, 1-way ANOVA with Dunnett’s test). (**J**) Representative images of GFP fluorescence (tumor cells) in tumor-implanted mice at 7dpi. Vessels were stained by DiD vessel paint (scale 25 µm). (**K**) Invading tumor cells marked by asterisks (scale 200 µm). Density of invading GFP-positive tumor cells ± SEM (*n* = 8–11, 1-way ANOVA with Dunnett’s test).

These findings prompted us to explore the possibility that moderate and simultaneous application of isoflurane might support drug transport into the CNS. Particularly in patients with glioblastoma, infiltrative tumor growth often occurs in the presence of an intact BBB.^31^ Hence, drugs with poor BBB permeability provide little therapeutic benefit.^1^ As a model system, we used GL261 glioma cells, a well-established tumor cell line that is isogenic to the C57BL/6 mouse strain and known for its aggressive growth. GL261 cells were implanted into the right caudate putamen and analyzed 14 days postimplantation (dpi). We first determined BBB permeability by EB extravasation in the tumor-bearing ipsilateral hemisphere as well as in the control contralateral hemisphere (**Figure 4A**). As 2.5% isoflurane for 30 min achieved the highest BBB permeability increase without causing edema, this optimal concentration was used in subsequent experiments. In agreement with our previous data, the intact contralateral hemispheres of mice treated with isoflurane achieved increased EB extravasation compared to control mice (**Figure 4B**). Untreated tumor-bearing ipsilateral hemispheres also showed increased BBB permeability compared to contralateral hemispheres, in agreement with tumor-mediated weakening of vessel tightness.^1^ Importantly, isoflurane further increased BBB permeability in tumor-bearing ipsilateral hemispheres, suggesting that accessibility of compounds to the tumor could be enhanced by isoflurane treatment.

We chose cisplatin (CP) as a proof-of-principle chemotherapeutic for our tumor model, as it poorly penetrates the CNS. CP affected viability of GL261 cells in vitro at concentrations as low as 2.5 µg/mL (**Figure 4C**). In contrast, GL261 cells were less sensitive by 2 orders of magnitude to temozolomide, the standard treatment of glioblastoma to date^32^ (Supplementary Figure 4A and B), rendering this drug inappropriate for our preclinical treatment paradigm. We implanted GL261 cells as described above. In a blinded and randomized 4-arm preclinical study, mice were treated twice, at 8 and 11 dpi, with 3 mg/kg CP and 2.5% isoflurane for 30 min or with sham conditions (CP only, saline, saline + isoflurane) (**Figure 4D**). All treatment regimens were well tolerated. Increased platinum content in brain sections as determined by LA-ICP-MS (laser ablation - inductively coupled plasma - mass spectrometry) suggested increased cisplatin content in cisplatin + isoflurane-treated mice compared to cisplatin-treated controls (Supplementary Figure 4C and D). When tumor volumes were quantified at 13 dpi by MRI, only the combination treatment with CP and isoflurane led to significantly reduced tumor growth (**Figure 4E and F**). We then determined a tumor disability index by calculating the ratio of dying (TUNEL positive) and proliferative (EdU positive, 16 h circulation time) tumor cells. Strikingly, the combination of CP and isoflurane significantly decreased tumor cell viability (**Figure 4G**, Supplementary Figure 4E and F). In contrast, in mice treated with CP tumor disability was comparable to saline controls.

Glioblastomas often show only limited intratumoral infiltration of immune cells. Tumor-associated macrophages, when polarized to an M2-like phenotype, suppress antitumor immunity.^31^ Microglia/macrophage infiltration was comparable in all treatment groups, whereas arginase-1-positive cells (a hallmark of the microglia/macrophage M2 signature) were slightly reduced following all treatments (Supplementary Figure 4G and H). When we quantified CD3-positive T lymphocytes in the tumor and the peritumor area, only the combination therapy with CP plus isoflurane revealed a significant increase in T cell density, thus suggesting a possible therapeutic benefit (**Figure 4H and I**). It is conceivable that the isoflurane-mediated enhanced BBB permeability not only facilitated cisplatin extravasation, but also improved T cell trafficking.

Tumor recurrence after standard therapy, comprised tumor resection, radiotherapy and chemotherapy, is caused by the highly invasive nature of glioblastoma cells.^33^ Invading tumor cells are often refractory to chemotherapeutics because of the largely impermeable BBB at sites of tumor cell migration.^1^ We observed here that implanted GL261 cells migrated along blood vessels traversing long distances (**Figure 4J**), well known from the human disease.^33^ We then explored whether the combination of CP and isoflurane affected these invading tumor cells. Strikingly, the number of tumor cells that had separated from the primary tumor was strongly reduced only in the CP plus isoflurane combination treatment group (**Figure 4K**). Together, these findings suggest that isoflurane co-application potentiated CP efficacy after only 2 treatment sessions, in as well as distant from the primary tumor, demonstrating a clear therapeutic advantage of this combination treatment.

## Discussion

An intact BBB is essential for normal brain function, also in brain diseases, when BBB damage can additionally affect tissue homeostasis.^2^ Here, we made the surprising finding that isoflurane exposure can be utilized to temporarily increase BBB permeability for CNS parenchymal drug delivery. When applied at moderate dosages, isoflurane did not damage brain cells, a finding which is in line with the low toxicity of this clinically approved and widely used anesthetic. Importantly, the BBB resealed at the end of short-term isoflurane exposure. These findings render the simultaneous application of neurological medication and volatile anesthetic a hopeful strategy for improved drug delivery in clinical application.

We show here that isoflurane facilitated brain import by means of caveolar transport. Caveolae assembly is suppressed under physiological conditions by the unique lipid environment in brain endothelial cells, despite the presence of all necessary biochemical components in brain capillaries.^20,21,26,34^ In model membranes, the hydrophobic isoflurane molecule dissolves in the bilayer and rapidly destabilizes especially compact liquid-ordered membrane regions by interfering with both hydrophobic membrane cores as well as glycerol backbones.^15,16^ The correlate of liquid-ordered domains in model membranes are membrane lipid rafts/lipid nanodomains in cellular membranes. We show that isoflurane enlarged the size of membrane lipid nanodomains in cultured primary brain endothelial cells. Together with the increased membrane fluidity in model membranes^14^ and altered regional distribution of membrane fluidity in brain vessels (this study), these findings support the concept that isoflurane facilitates the local reorganization of nanodomain lipids that favors spontaneous formation of caveolar invaginations overruling the otherwise inhibitory lipid environment (Supplementary Figure 5).

The destabilization of the compact lipid order could also directly enhance diffusion across the endothelial membrane bilayers. It is possible that this caused the vasogenic edema and hypoxia signaling in the absence of neuroinflammation that we observed after prolonged 2.5% isoflurane treatment in mice. Despite the almost 180-fold increased BBB permeability observed after this isoflurane protocol, tight junction morphology was unaffected. This situation resembles early events after stroke, when BBB impairment occurs despite intact endothelial tight junctions^22^ and highlights the importance of modest isoflurane dosing.

In a proof-of-concept preclinical trial, the simultaneous application of a prototypic cytotoxic drug and isoflurane improved chemotherapeutic efficacy in a murine brain tumor model by affecting tumor cell viability. In addition to improving accessibility of the cytotoxic, isoflurane enhanced immune cell infiltration, likely by facilitating caveolar transport as in an inflammatory model of multiple sclerosis.^35^ In cancer patients, isoflurane exposure could also directly attenuate tumor cell proliferation by impairment of mitochondrial respiration,^36^ as in mouse mutants with defective complex I of the respiratory chain.^37^ On the other hand, activated HIF signaling could lead to enhanced tumor cell proliferation, angiogenesis, recruitment of tumor-associated macrophages, and drug resistance.^1^ However, isoflurane administration alone (without chemotherapy) did not alter any measures of tumor growth and viability in our murine glioblastoma model. These findings emphasize the significance of simultaneous administration of isoflurane and chemotherapy.

At present, the most promising methods to overcome the BBB in therapy of brain tumors are focused ultrasound, inhibition of ABC efflux transporters and the administration of drugs coupled to various types of nanocarriers.^1,25^ While focused ultrasound likely achieves strong and spatially precise BBB breaches by the mechanical force of microbubble oscillation, the advantage of the combined isoflurane/drug treatment is the broad drug delivery, including to infiltrating tumor cells and small brain metastases. Strategies to design nanocarriers and to inhibit ABC transporters have greatly advanced in recent years, particularly through research on glioblastoma therapies.^1,32^ It is conceivable that transport of these drug vehicles could benefit from utilizing caveolar transcytosis, which can be achieved by co-application of inhalation anesthetics. The isoflurane-mediated drug delivery in mice provides a starting point for the development of therapeutic strategies for a broad spectrum of neurological diseases, and translational studies are necessary to determine the feasibility in patients.

## Supplementary Material

vdab140_suppl_Supplementary_Figure_S1

vdab140_suppl_Supplementary_Figure_S2

vdab140_suppl_Supplementary_Figure_S3

vdab140_suppl_Supplementary_Figure_S4

vdab140_suppl_Supplementary_Figure_S5

vdab140_suppl_Supplementary_Materials
