# Supplementary figures and images for: Anesthesia triggers drug delivery to experimental glioma in mice by hijacking caveolar transport
Source: Neurooncol Adv. 2021 Sep 20;3(1):vdab140. doi: 10.1093/noajnl/vdab140 (PMC8500692; doi:10.1093/noajnl/vdab140)

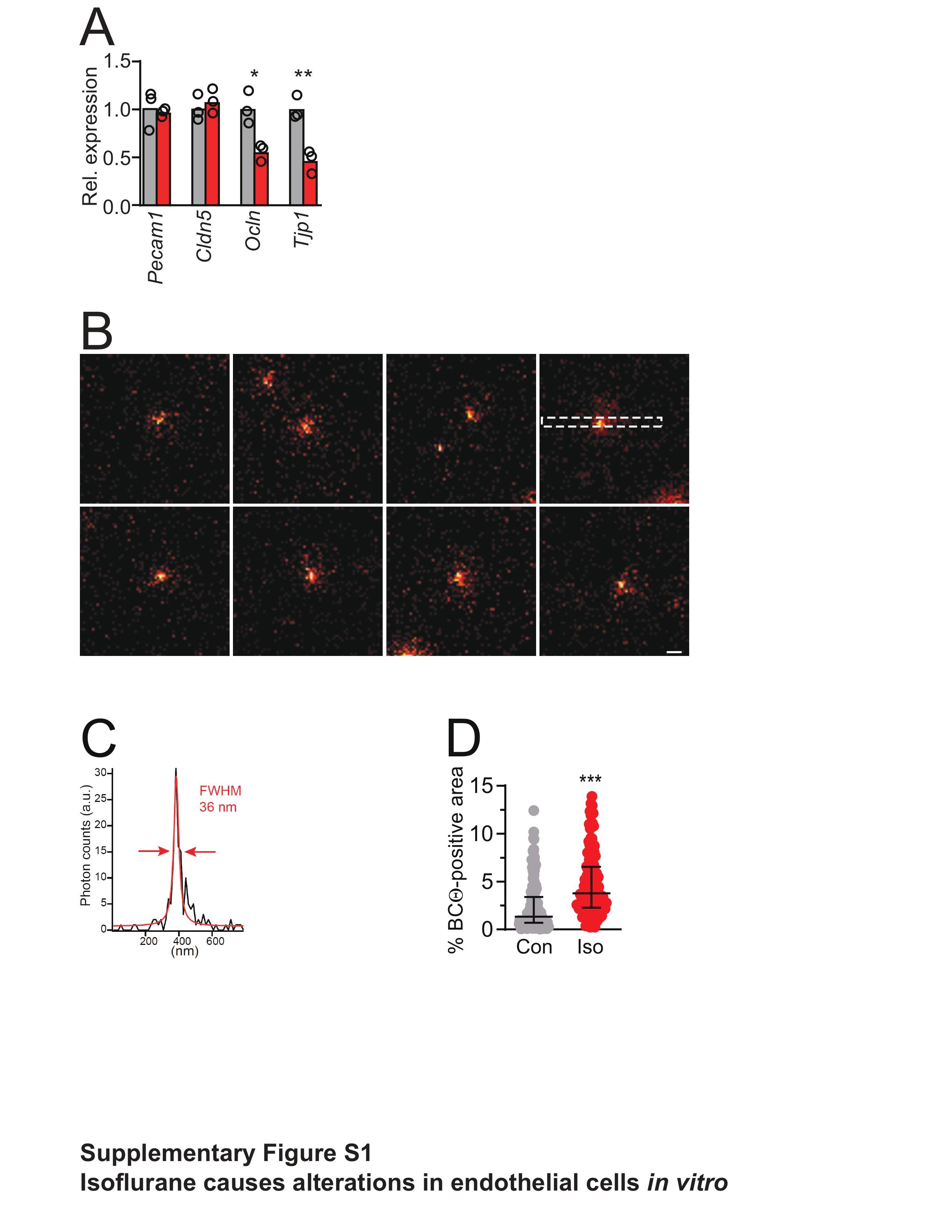

Supplement: vdab140_suppl_Supplementary_Figure_S1 [file vdab140_suppl_supplementary_figure_s1.jpeg]

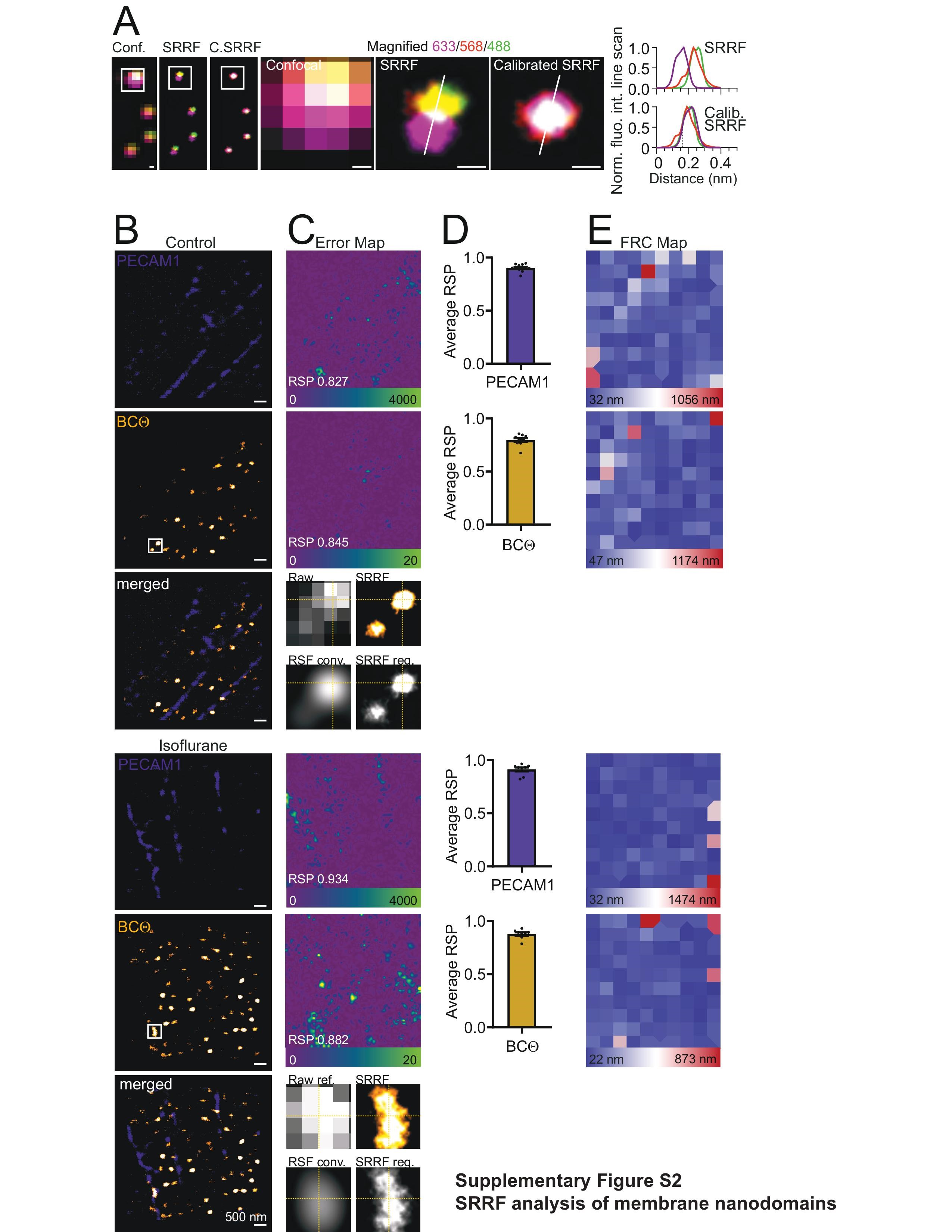

Supplement: vdab140_suppl_Supplementary_Figure_S2 [file vdab140_suppl_supplementary_figure_s2.jpeg]

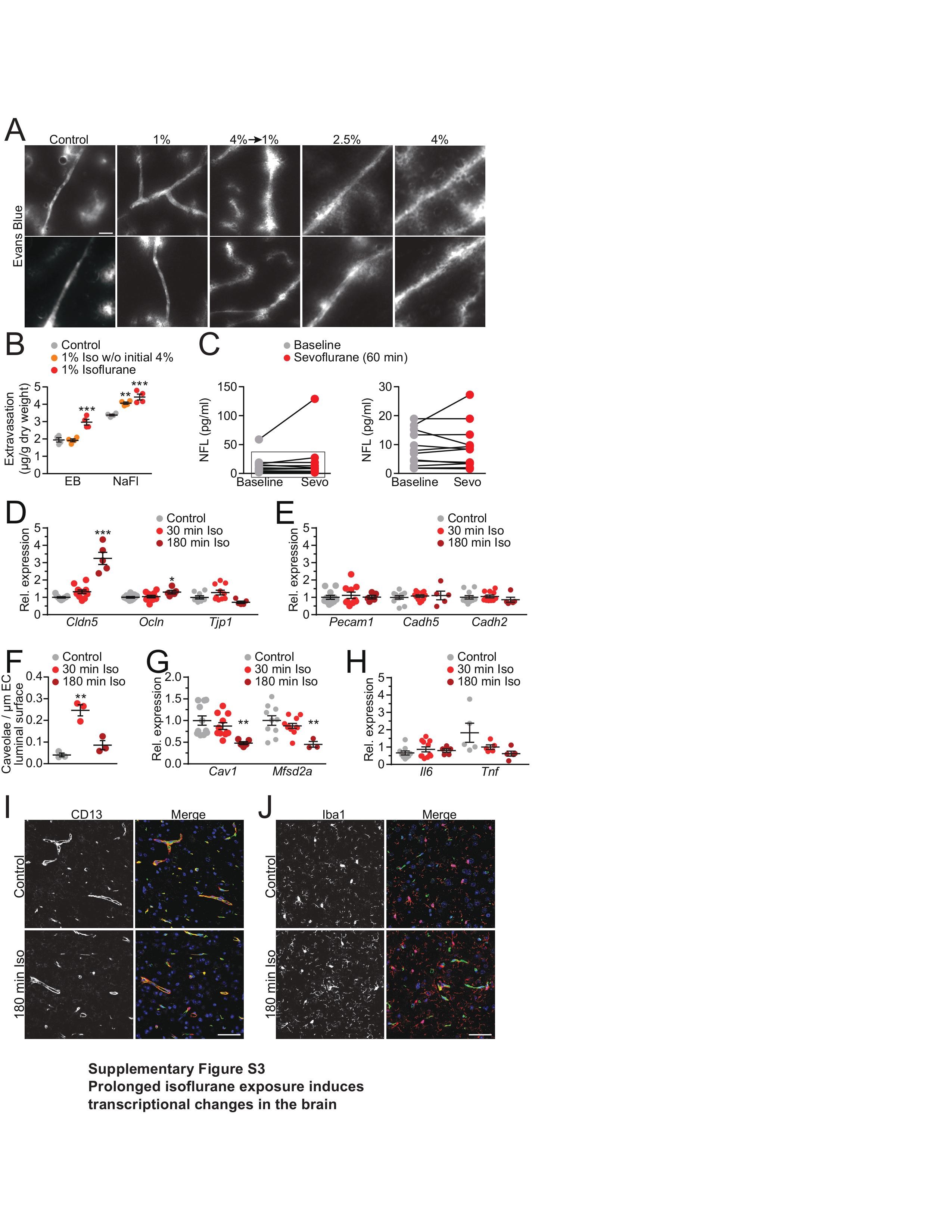

Supplement: vdab140_suppl_Supplementary_Figure_S3 [file vdab140_suppl_supplementary_figure_s3.jpeg]

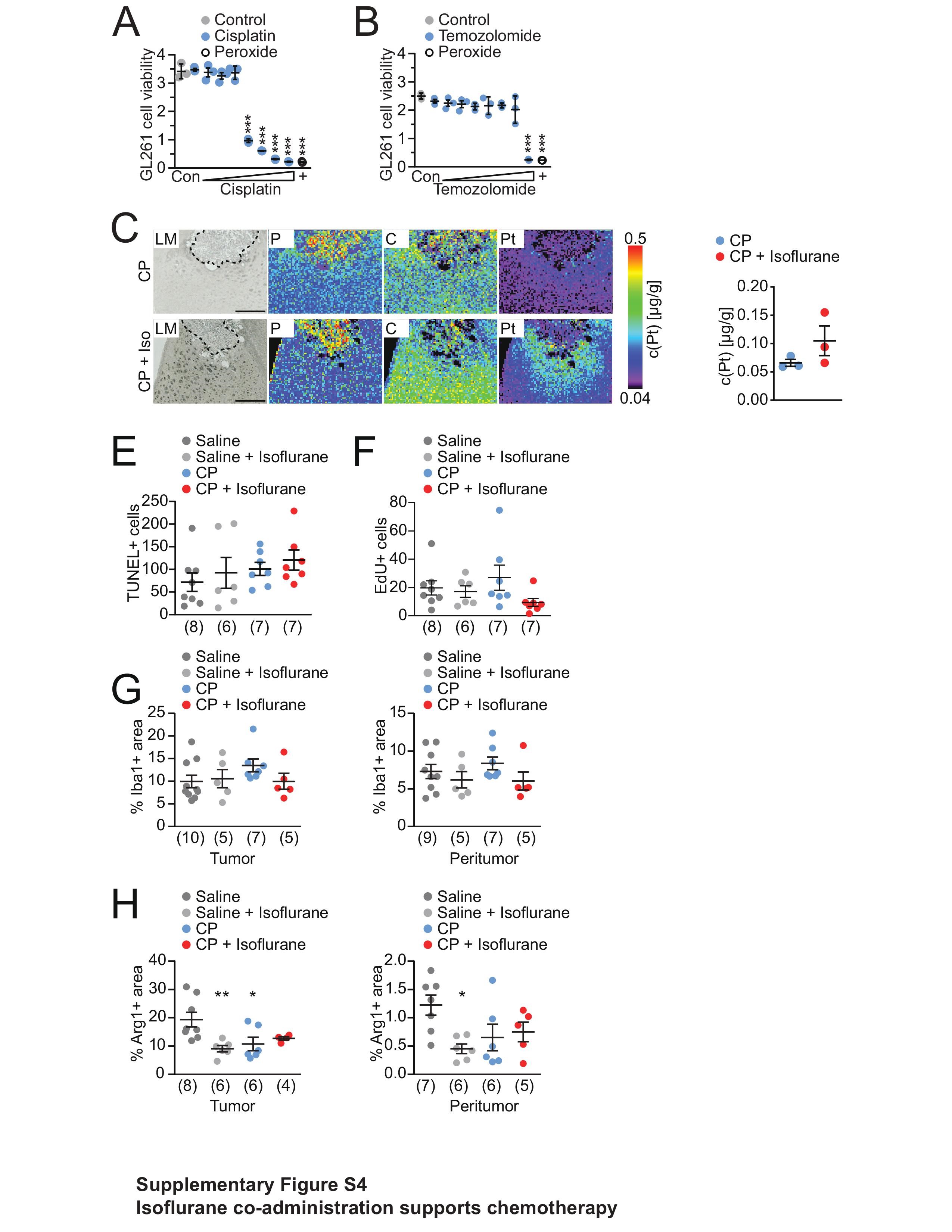

Supplement: vdab140_suppl_Supplementary_Figure_S4 [file vdab140_suppl_supplementary_figure_s4.jpeg]

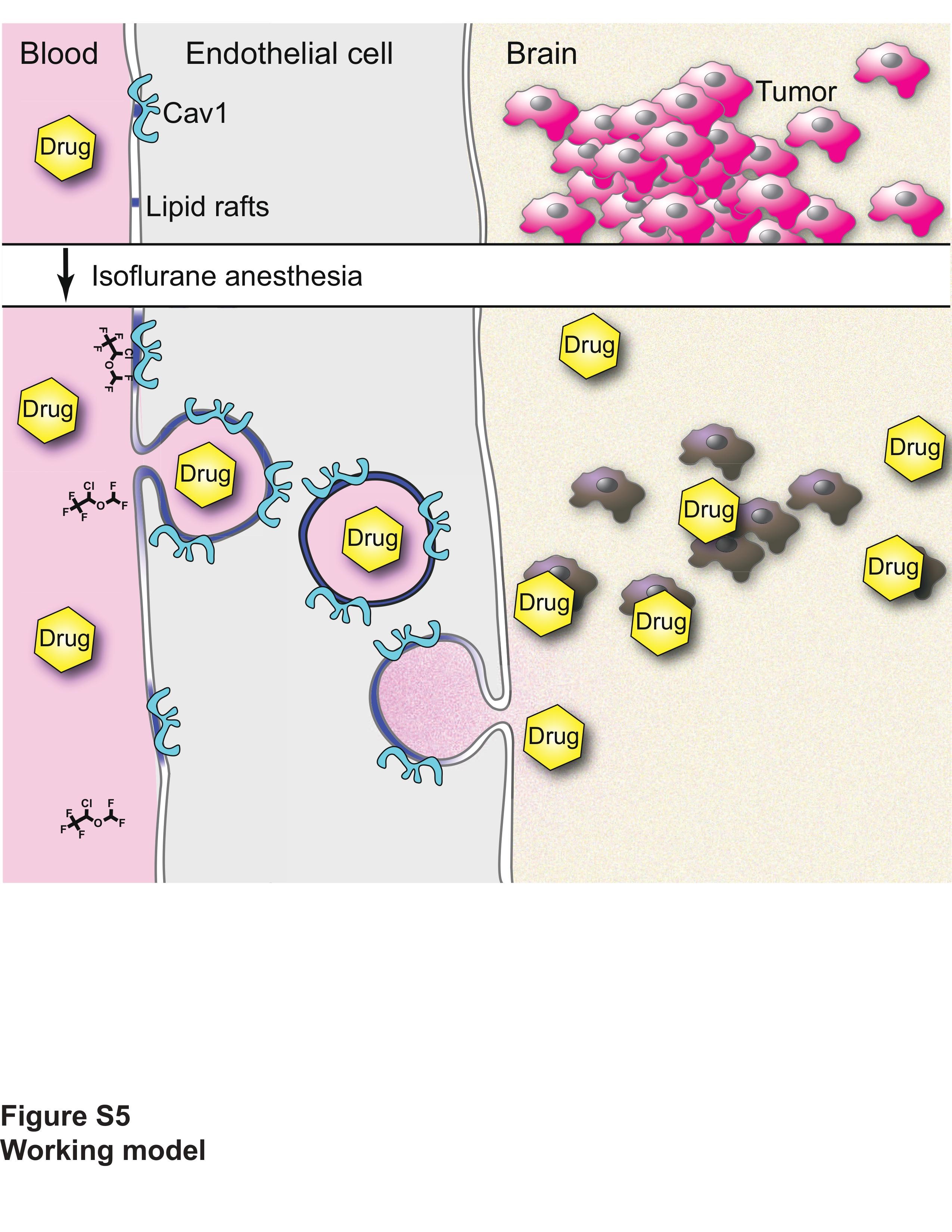

Supplement: vdab140_suppl_Supplementary_Figure_S5 [file vdab140_suppl_supplementary_figure_s5.jpeg]
