# Supplementary material for: Anesthesia triggers drug delivery to experimental glioma in mice by hijacking caveolar transport
Source: Neurooncol Adv. 2021 Sep 20;3(1):vdab140. doi: 10.1093/noajnl/vdab140 (PMC8500692; doi:10.1093/noajnl/vdab140)
Supplement: vdab140_suppl_Supplementary_Materials [file vdab140_suppl_supplementary_materials.docx]

Supplementary Material

Anesthesia triggers drug delivery to experimental glioma in mice by hijacking caveolar transport

Lena Spieth, Stefan A. Berghoff, Sina K. Stumpf, Jan Winchenbach, Thomas Michaelis, Takashi Watanabe, Nina Gerndt, Tim Düking, Sabine Hofer, Torben Ruhwedel, Ali H. Shaib, Katrin Willig, Katharina Kronenberg, Uwe Karst, Jens Frahm, Jeong Seop Rhee, Susana Minguet, Wiebke Möbius, Niels Kruse, Christian von der Brelie, Peter Michels, Christine Stadelmann, Petra Hülper and Gesine Saher*

LS, SAB, and SKS contributed equally to this work.

Supplementary Methods

Supplementary Figure 1. Isoflurane causes alterations in endothelial cells in vitro.

Supplementary Figure 2. SRRF analysis of membrane nanodomains.

Supplementary Figure 3. Long-term isoflurane induces transcriptional changes in the brain.

Supplementary Figure 4. Isoflurane co-administrations supports chemotherapy.

Supplementary Figure 5. Working model.

Supplementary Table

Additional supplementary references

Supplementary Methods

### Animals

All animal experiments were performed in compliance with the ARRIVE guidelines and animal policies of the Max Planck Institute of Experimental Medicine, and were approved by the German Federal State of Lower Saxony (Lower Saxony State Office for Consumer Protection and Food Safety). Male C57BL/6N WT and caveolin-1 deficient^38^ mice were bred and kept in individually ventilated cages with a 12-hour day/night cycle and used at 8-10 weeks of age. Only male mice were included in this study. Animals were randomly assigned to the different treatment groups. Caveolin-1 deficient mice were compared to littermate controls. Mice were put into the isoflurane evaporator chamber (Isotek5, Groppler) preflushed with 4% isoflurane for 2 min unless otherwise indicated, and then with isoflurane at indicated dosage for another 28 min or 178 min at a flow rate of 0.7 l/min with oxygen as carrier gas. Sevoflurane was applied at a concentration of 8% for 2 min, followed by 2.5% or 4.5% for 28 min. Body temperature and respiration was controlled during anesthesia (Animal temperature control ATC1000). Mice were allowed to awaken with oxygen at the end of anesthesia.

### Patients

Ethical approval for was obtained from the ethics committee of the University Medical Center (#19/5/20). Informed consent was obtained from all patients. To analyze, whether general anesthesia with sevoflurane results in a similar increase in BBB disruption, serial blood samples were obtained from neurosurgical patients. Only patients with degenerative spinal diseases such as lumbar disc prolaps or degenerative lumbar canal stenosis were included as we assumed that their BBB was intact. The first blood sample was taken directly before the induction of general anesthesia. Then, general anesthesia was initiated with propofol bolus injection (1.5 mg/kg body weight) and maintained using age-adapted 0.8-1 MAC sevoflurane with oxygen as carrier gas. The second blood sample was obtained 1 hour after the induction of the general anesthesia before commencing the surgery. Serum samples were stored at -80°C and analyzed after all specimens were collected in order to rule out methodological differences over time. Surgical treatment and clinical course were uneventful in all patients.

### Cell culture

Primary mouse brain EC cultures were established from 7 days old mice or rats as described^9^. Briefly, cortices were digested with 1 mg/ml collagenase/dispase and 2.5 μg/ml DNAse (Roche) in dissection buffer (HBSS, 10mM HEPES, 0.5% BSA, 5000 U/ml penicillin/streptomycin) for 45 min at 37°C. After trituration, cells were resuspended in 25% BSA and centrifuged at 1000 g for 20 min to pellet microvessels. Isolated microvessels from individual mice were plated in Endobasal Medium (Promocell) with 0.4% puromycin for positive selection on coverslips or polyester transwell inserts (Corning). Primary astrocyte cultures were prepared from 0 to 2 days old mice as described^9^. For triple culture experiments^39^, ECs cultured in transwell inserts as described above, and astrocytes were plated on the bottom of the well plate. Primary pericytes were cultured on the bottom side of the well. Media were treated with indicated concentrations of isoflurane gas in oxygen or vehicle in a gas chamber for 30 min. Confluent cell cultures were incubated with isoflurane-fumigated media for 30 min or treated with 10 mM methyl-beta-cyclodextrin (Sigma). An epithelial Voltohmmeter (EVOM2, World Precision Instruments) equipped with Endohm-12 chamber electrodes was used to measure transendothelial electrical resistance (TEER). GL261 tumor cells^40^ were treated with Cisplatin or temozolomide, the standard treatment of glioblastoma to date^32^. Metabolic activity / viability was determined by WST1 assays (Cayman) that quantifies enzymatic activity of mitochondrial dehydrogenases present in viable cells, according to the manufacturer’s protocol. As a positive control of impaired viability, 20 µM peroxide was applied. Cells were fixed with 4% paraformaldehyde and processed for immunofluorescence analysis. Biotinylated BCtheta was used as previously described^41^ at 0.015 mg/ml and visualized with streptavidin-coupled Abberior STAR 635P (Abberior) for STED microscopy or with streptavidin-coupled Alexa488 for confocal imaging for subsequent SRRF analysis.

### Super-resolution microscopy

STED microscopy was performed with a custom-built STED microscope as described^42^ (**Supplementary Figure 2**). Abberior STAR635 was excited at 650 nm with 17 µW and stimulated depletion was performed at 775 nm with a power of 560 mW at the back aperture of the objective lens. Line scan SRRF acquisitions were carried out using a 12 KHz resonant scanner from Leica TCS SP8 lightning confocal microscope (**Supplementary Figure 3**). The cells were excited with 10% white light laser at 561 and 488 nm with image acquisition settings as described^43^. 3000 frames with a region of 6.4 X 6.4 µm and a pixel size of 100 nm were acquired using HyD detectors in counting mode. SRRF analysis was based on a NanoJ-SRRF plugin. Drift correction was always applied and chromatic aberration was accounted for using 100 nm Tetraspeck beads that were acquired and resolved by using the SRRF method. SRRF input parameters involved a ring radius of 0.5, radiality magnification 10, axes in ring 8, time lapse 0, crop data 0, max temporal analysis block size 100, preferred spatial block size 0, temporal analysis TRAC, TRAC order 4, gradient smoothing activated, weighting default, PSF FWHM 3.17 and corrections default. Resolution and error maps were calculated using NanoJ-SQUIRREL plugin with default settings^44^. The analysis was automated using our own specifically programmed macro. For quantification of super-resolution images, three 4x4 µm regions of interest per cell were selected in maximum intensity plots of image stacks. The area of the nanodomains was determined in thresholded images with a size exclusion of 16 pixels (3600 nm^2^) using the Fiji particle analyzer plugin^45^.

### Histochemistry

Mice were sacrificed by cervical dislocation and immersion-fixed for 48 h or perfused with 4% formaldehyde (PFA). For marker independent staining of the vasculature by vessel paint, DiI or DiD was included in the perfusion procedure as described^9^. Tissue blocks were embedded in paraffin and cut into 5 µm sections (HMP 110, MICROM). Sections were deparaffinized followed by antigen-retrieval in sodium citrate buffer (0.01 M, pH 6.0). For immunolabeling, sections were blocked with serum-free protein block (Dako). Primary antibodies were diluted in 2% bovine serum albumin (BSA)/PBS and incubated for 48 h. Detection was carried out using fluorophore-coupled secondary antibodies, the LSAB2 kit (Dako) or the Vector Elite ABC kit (Vector Labs). HRP substrate 3,3'-diaminobenzidine was applied by using the DAB Zytomed Kit (Zytomed Systems GmbH). Haematoxylin stain was performed to label nuclei. Sections were dehydrated prior to mounting (Eukitt). Specimens were analyzed on an Axio Imager.Z1 (Zeiss) equipped with an AxioCam MRc3, 0.63x Camera Adapter and the ZEN 2012 blue edition software using a 10x objective (Plan Apochromat 10x/0.45 M27) or a 20x objective (Plan-Apochromat 20x/0.8) and evaluated with Image J software. Three to five sections per animal were analyzed for quantification (CD3 positive T cells). For the EdU/TUNEL assay, slides were incubated with proteinase K (20 μg/ml) for 10 min, followed by consecutive labeling of dying cells by TUNEL (Promega) and of proliferating cells by EdU click-it labeling (Thermo Fisher), according to the manufacturers’ instructions.

Electron microscopic analysis was done as described^4^. Briefly, tissue was fixed in 4% PFA, 2.5% glutaraldehyde, 0.1 M phosphate buffer. Sagittal brain sections were cut on a vibratome (Leica VT1200, 300 µm) and the striatum was punched with a 2 mm diameter punching tool. Tissue punches were embedded in epon (LynxII, EMS). Of each of the 3 mice per treatment group, at least 15 digital pictures using an on-axis 2048x2048-CCD camera (12000× magnification, TRS, Moorenweis) of uranyl acetate contrasted ultrathin sections were taken with the LEO912 electron microscope (Zeiss, Oberkochen).

For analysis of generalized polarization (GP) analysis of Laurdan fluorescence, mice were treated with or without 2.5% isoflurane for 30 min, and Laurdan (6-Dodecanoyl-2-Dimethylaminonaphthalene, Sigma) vessel paint was included in the perfusion procedure. After postfixation in PFA overnight, brains were cut in 200 µm sagittal sections on a vibratome and vessels were imaged by 2Photon microscopy (LaVision) and processed as described^17^.

### Protein analysis

Tissue samples were lysed in sucrose buffer (18% sucrose, 10 mM Tris/HCl pH 7.4, 1 mM sodium bicarbonate, 1 mM magnesium chloride, 0.1% Triton, 0.2% lithiumdodecyl sulphate, 0.025% sodium deoxycholate) with protease inhibition (Roche) using a Precellys 24 homogenizer (Bertin technologies). SDS gel electrophoresis, semi-dry blotting on PVDF membranes (Hybond P, Biosciences) and antibody incubations were done using standard procedures. Detection of immunolabeled proteins was performed with Western-Lighting Plus-ECL Reagent (Perkin Elmer) using ChemoCam Imager (Intas). Proteins were quantified by normalization to reprobed tubulin signal.

### Expression analysis

Expression analyses were carried out as described^9^. RNA was extracted from dissected tissue using QIAshredder and RNeasy protocols (Qiagen). Concentration and quality of RNA were evaluated using a NanoDrop spectrophotometer and RNA Nano (Agilent). cDNA was synthesized with Superscript III (Invitrogen) and quantitative PCRs were done in triplicate with the GoTaq qPCR Master Mix (Promega) on a 7500 Fast Real-Time PCR System (Applied Biosystems). Expression values were normalized to the mean of two housekeeping genes, HPRT (hypoxanthin-phosphoribosyl-transferase 1) and Rplp0 (60S acidic ribosomal protein P). Quantification was carried out using the ΔΔCt method, normalized to age matched untreated controls (set to 1). All primers were intron-spanning. Expression of the following genes was measured: *Aqp4* (aquaporin-4) *Cldn5* (claudin 5), *Ocln* (occludin), *Pecam1* (CD31), *Tjp1* (ZO1, zona occludens protein 1), *Egln1* (Egl-9 family hypoxia-inducible factor 1 encoding hypoxia-inducible factor prolyl hydroxylase 1), *Egln2, Egln3,* *Vegfa* (vascular endothelial growth factor A), *Tgfb3* (transforming growth factor beta-3). All primer sequences are listed in the Supplementary Table.

### Blood-brain barrier permeability

Measurements of BBB permeability was done as described^3,9^. Briefly, tracers were i.v. injected (Evans blue 50 mg/g body weight). Afterwards mice were immediately put into the isoflurane evaporator chamber (Isotek5, Groppler) preflushed with the desired isoflurane concentration at a flow rate of 0.7 l/min. Body temperature and respiration were controlled (animal temperature control ATC1000) during anesthesia and at the end of isoflurane treatment the mice were awaken with 100% oxygen. For quantification of tracer extravasation, animals were perfused with PBS to remove tracer from the circulatory system. The region of interest was isolated and tissue samples were weighed. Tissue samples were lyophilized at a shelf temperature of –56 °C for 24h under vacuum of 0.2 mBar (Christ LMC-1 BETA 1-16) and extracted with formamide at 57°C for 24h on a shaker at 300 rpm. Integrated density of tracer fluorescence was determined in triplicates after 1:3 ethanol dilutions to increase sensitivity. Tracer concentration was calculated using a standard curve prepared from tracer-spiked brain samples.

### Tumor cell implantation and anti-tumor treatment

Eight weeks old mice were anesthetized with ketamine/xylazine. Using a stereotactic instrument (Kopf), 7.5 x10^4^ GL/261-GFP cells were injected 2 mm lateral, 1 mm anterior and 3 mm deep into the right hemisphere of mice. The wound was sealed with tissue glue (Histoacryl) and 4 mg/kg of the analgetic carprofen was injected subcutaneously. Respiration and body temperature was constantly monitored until the mice woke up and recovered from anesthesia. Mice were randomly assigned to one of four groups: cisplatin, cisplatin + isoflurane, saline and saline + isoflurane. We chose cisplatin (CP) as proof-of-principle chemotherapeutic for our tumor model, as it poorly penetrates the CNS^46^. At days 8 and 11 after implantation, mice were treated with i.v. 3 mg/kg cisplatin (0.5 mg/ml cisplatin in saline 0.9% NaCl) or an equal volume of saline. Afterwards mice were immediately treated with isoflurane (4% isoflurane for 2 min and then with 2.5% isoflurane for another 28 min). Due to nephrotoxic side effects of the cisplatin treatment, mice received three saline injections before and after the treatment, as well as after 24h. A single i.p. injection of EdU was applied 16 h before the mice were sacrificed on day 14 after implantation.

### MRI

Animals were initially anesthetized using a chamber pervaded with 5% isoflurane. Subsequently, the mice were intubated, artificially ventilated and maintained under anesthesia with 1.75% isoflurane in ambient air. MRI was performed at 9.4 T (Bruker Biospin MRI GmbH, Ettlingen, Germany). Radiofrequency excitation was carried out using a birdcage resonator (inner diameter, 72 mm), and signals were received using a four-channel phased-array surface coil. T2-weighted MRI data were acquired with a three-dimensional fast spin-echo MRI sequence (repetition time TR = 3.5 s, effective echo time TEeff = 55 ms, 12 differently phase-encoded echoes, 56 minutes measuring time) at an isotropic spatial resolution of 100 µm. The resulting DICOM datasets were imported into Amira^TM^ (Amira Software, Visage Imaging, Germany) for 3D reconstruction and calculation of the tumor sizes. The region of interest was manually as well as semi-automatically labelled and tumors were segmented on three-dimensional label fields (80 horizontal, 192 coronal, and 144 sagittal slices). Analyses were performed blinded to the treatment.

### Laser ablation - inductively coupled plasma - mass spectrometry (LA-ICP-MS)

To quantify cisplatin delivery to the brain, platinum was spatially localized and quantified by elemental bioimaging by means of laser ablation – inductively coupled plasma – mass spectrometry (LA-ICP-MS) on brain sections from mice treated with CP or CP plus isoflurane essentially as described^47^.

For elemental bioimaging by LA-ICP-MS analysis, 5 µm thin sections of the brain tissue were dewaxed (60°C for 10 min, xylene for 10 min, decreasing ethanol concentrations 100%, 90% and 80% for 5 min each and ddH_2_O for 2 min). Microscopic images were recorded using a BIOREVO BZ-9000 digital microscope (Keyence, Osaka, Japan). For the quantification of Pt, four matrix-matched standards based on gelatine (10% w/w) including a blank standard were enriched with Pt concentrations ranging from 1 to 10 μg/g and cut into 5 µm thin sections. An LSX 213 G2+ laser system (CETAC Technologies, Omaha, NE, USA) was equipped with a two-volume HelEx II cell and hyphenated to an Agilent 7700 Series ICP-MS (Agilent Technologies, Santa Clara, CA, USA). The ablation was performed in a line-by-line scan with a spot size of 25 µm, a scan speed of 50 µm/s and He as transport gas with a flow of 800 ml/min. The ICP-MS was operated in the kinetic energy discrimination mode with a He gas flow of 1.5 ml/min and a H_2_ gas flow of 1.0 ml/min. An integration time of 45 ms was set for the isotopes ^13^C and ^31^P and 300 ms for ^195^Pt. For the external calibration, averaged intensities of the scanned lines of the standards were evaluated by a weighted linear regression using the weighting factor 1/x_i_^2^ and confirmed sufficient linearity with a regression coefficient of R^2^ ≥ 0.998 over the analysed concentration range. Limit of detection (LOD) and limit of quantification (LOQ), calculated with the 3σ- and 10σ-criteria, were 0.04 µg/g and 0.12 µg/g for Pt. The quantification, visualization and region of interest (ROI) analysis were performed with the in-house developed software ImaJar (by Robin Schmid, WWU Münster, Münster, Germany). Averaged Pt concentrations of ROIs were calculated by excluding pixels without tissue and thus below the background signal of the isotope ^13^C.

Supplementary Figure Captions

Supplementary Figure S1. **Isoflurane causes alterations in endothelial cells *in vitro*.**

(**A**) Relative mRNA expression of genes as indicated in primary endothelial cells in response to 30 min treatment with 1% isoflurane followed by media change and analysis after 24 h (n=3 independent samples, Student’s t-test, * P<0.05, ** P<0.01).

(**B**) To estimate the resolution of STED images, we selected 8 small clusters and averaged 3 horizontal lines over each cluster to increase signal to noise ratio. The averaged fluorescence signal was fitted with a Lorentzian function to determine the full-width at half-maximum (FWHM) with a pixel size of 10 x 10 nm (scale 100 nm).

(**C**) Example line profile (black) and Lorentzian fit (red) with FWHM (arrows) of marked area in (B). The average FWHM (± SD) was 35.88 ± 9.69 nm, and thus 36 nm was taken as upper limit of the resolution.

(**D**) Percent of BCtheta-positive area in the plasma membrane of cells treated with or without isoflurane, shown as median ± interquartile range (n = 50-51 cells from 3 experiments, Mann-Whitney test).

Supplementary Figure S2. **SRRF analysis of membrane nanodomains.**

(**A**) Chromatic aberration in SRRF-generated images was corrected using 100 nm tetraspeck beads excited with 633 (magenta), 561 (red) and 488 nm (green). A merged maximum intensity projected confocal image (conf.), is converted to a SRRF-resolved image (SRRF) which is then calibrated by correction of the chromatic aberration (C.SRRF), with boxed detail on the right. Normalized fluorescence intensity line scans across a single bead from SRRF and calibrated SRRF images were used for alignment of the bead position across the three different channels. The channel correction coordinates of beads were applied to the SRRF-generated data using the same excitation light sources and acquisition parameters (100 x 100 nm, 3000 frames acquired at 12 KHz, 6.4 µm x 6.4 µm and drift correction, scales 100 nm).

(**B**) Representative calibrated SRRF images of PECAM1 and BCθ of control and isoflurane-treated cells.

(**C-E**) SQUIRREL analysis was performed to ensure minimal artefacts in our current acquisition settings. Super-resolved images were generated by calculating error maps and resolution-scaled Pearson’s coefficients (RSPs) as described ^41^. (**C**) In the comparison between the SRRF-resolved image and the reference confocal image, purple color indicates agreement between the images and yellow as well as green disagreement. The smaller images below visualize the process of calculating the error map. The NanoJ-SRRF plugin uses the reference raw image (upper left) and the convolved super-resolution image for approximating the resolution scaling function (RSF). The software identifies any differences in pixel magnification and size between the two images and plots it in an error map. The upper right image shows SRRF resolved image, the lower left image is convolved with RSF and the lower right is the registered calculated SRRF image. (**D**) Average RSP calculation revealed for control PECAM1 and BCθ 9.133 and 0.8784, respectively (n=8) and for the isoflurane-treated cells, the RSP of PECAM1 and BCθ was 0.902 and 0.07973, respectively (n=11). (**E**) Fourier ring correlation (FRC) map was plotted for each of the data points as a color map representation, with blue color indicating lower FRC values (higher resolution), and red indicating higher FRC values (lower resolution). A non-square shaped block can be generated in the process. This denotes the areas in which correlations between the two source images at these coordinates were insufficient to calculate the FRC value. To calculate FRC values, FRC information is tessellated from neighboring blocks. The average lower resolution for the two channels was less than 50 nm in all conditions.

Supplementary Figure S3. **Prolonged isoflurane exposure induces transcriptional changes in the brain.**

(**A**) Representative images of isoflurane induced Evans blue extravasation. Mice were i.p. injected with Evans blue and treated with increasing doses of isoflurane for 30 minutes as indicated, after which mice were sacrificed and Evans blue fluorescence was imaged in acute brain sections (scale 10 µm).

(**B**) Mean relative tracer extravasation (Evans blue or sodium fluorescein) in response to 1% isoflurane for 30 minutes with or without initial exposure to 4% isoflurane for 2 minutes +/-SEM (n = 4 mice, 1way ANOVA with Dunnett’s test in comparison to untreated control cultures). Treatment without initial 4% isoflurane exposure does not cause deep anesthesia.

(**C**) Single molecule array of serum NFL in human samples (n=13) before and after a 60 min exposure to sevoflurane (0.8-1 MAC). Except for one patient, NFL values remained largely unchanged.

(**D-E**) Mean mRNA expression of (**D**) endothelial tight junction proteins *Cldn5* (claudin 5), *Ocln* (occludin), *Tjp1* (ZO1), (**E**) other endothelial junction proteins *Pecam1* (platelet and endothelial cell adhesion molecule 1), *Cadh5* (cadherin 5), *Cadh2* (cadherin 2) ± SEM in subcortical brain tissue of control mice or mice treated with 2.5% isoflurane for 30 or 180 min (n=5-11 mice, 1way ANOVA with Dunnett’s post test for each gene, indicated are significant changes to control animals).

(**F**) Mean density of luminal micro-invaginations (caveolar profiles) ± SEM in electron micrographs from mice exposed to isoflurane and controls (39 and 41 capillaries from n=3 animals, Student’s t-test).

(**G-H**) Mean mRNA expression of (**G**) transcytosis proteins caveolin (*Cav1*) and major facilitator superfamily domain containing 2a (*Mfs2da*), (**H**) pro-inflammatory markers *Il6* (interleukin 6), *Tnf* (tumor necrosis factor) ± SEM in subcortical brain tissue of control mice or mice treated with 2.5% isoflurane for 30 or 180 min (n=5-11 mice, 1way ANOVA with Dunnett’s post test for each gene, indicated are significant changes to control animals).

(**I-J**) Representative images of brain sections from mice treated with 180 min isoflurane or left untreated, stained in red for (**I**) pericytes (CD13) or (**J**) microglia (Iba1) together with vessels (IB4-lectin, green) and nuclei (DAPI) (scale 50 µm).

* P<0.05, ** P<0.01, *** P<0.001

Supplementary Figure S4. **Isoflurane co-administrations supports chemotherapy.**

(**A, B**) Mean viability of GL261 tumor cells ± SEM that were treated with increasing concentrations of (**A**) cisplatin (0, 0.025, 0.05, 0.25, 0.5, 2.5, 5, 25, 50 µg/ml) or (**B**) temozolomide (0, 0.5, 2.5, 5, 25, 50, 100, 250, 500 µg/ml) or peroxide (2 mmol) for 72h measured by a WST1 assay (n=3, 1way ANOVA with Dunnett’s comparison to control conditions).
(**C-D**) Representative images (**C**) and quantification (**D**) of platinum (Pt) in tissue sections from tumor-bearing mice euthanized at 14 dpi that had received chemotherapy with CP alone or CP + isoflurane at 8 and 11 dpi (n=3 mice). Platinum concentrations were determined relative to standards by LA-ICP-MS. Carbon (C) and phosphorous (P) levels are provided as relative signal intensity plots. The tumor outline (dashed line) is marked on the light microscopic image (LM; scale, 0.5 mm).
(**E-F**) TUNEL+ cells (**E**) and EdU+ cells (**F**) per mm^2^ tumor area. Depicted are mean values with individual data points ± SEM (n=6-8 animals as in parenthesis).
(**G-H**) Quantification of the relative immunostained area in the tumor area (left panels) or the peritumor area (right panels) immunostained for all microglia (Iba1, **G**) or M2-type arginase-1 expressing microglia (Arg1, **H**). Depicted are mean values with individual data points ± SEM (n=4-10 animals as in parenthesis, 1way ANOVA with Dunnett’s post test).

* P<0.05, ** P<0.01, *** P<0.001

Supplementary Figure S5. **Working model.**

Despite the presence of all necessary biochemical components in brain capillaries, caveolae assembly is suppressed under physiological conditions by the unique lipid environment in brain endothelial cells^20,21,26,34^. We hypothesize that isoflurane exerts a small interference on the lipid order, e.g. by locally increasing membrane fluidity allowing the spread of cholesterol out of the core of lipid nanodomains. This could destabilize the suppressive function of the local lipid composition around nanodomains. The local enrichment of nanodomain lipids could then favor spontaneous formation of caveolar invaginations overruling the otherwise inhibitory lipid environment leading to import and export of caveolar cargo. In contrast, endothelial tight junctions remain intact. Within the brain, isoflurane-mediated drug import affected viability of cells in the tumor mass and additionally invading tumor cells.

Supplementary Table

### Primer sequences used in expression analyses.

Primers used for expression analysis were intron-spanning (5’-3’).

| Mouse |  |
| --- | --- |
| Hprt | TCCTCCTCAGACCGCTTTT - CCTGGTTCATCATCGCTAATC |
| Rplp0 | GATGCCCAGGGAAGACAG - ACAATGAAGCATTTTGGATAATCA |
| Cldn5 | ACGGGAGGAGCGCTTTAC - GTTGGCGAACCAGCAGAG |
| Ocln | TCCGTGAGGCCTTTTGAA - GGTGCATAATGATTGGGTTTG |
| Pecam1 | GCTGGTGCTCTATGCAAGC - ATGGATGCTGTTGATGGTGA |
| Tjp1 | ATGCAGACCCAGCAAAGGT - TGACCAAGAGCTGGTTGTTTT |
| Egln1 | GGAACCCACATGAGGTGAA - AACACCTTTCTGTCCCGATG |
| Egln2 | CTGTGGAACAGCCCTTTTTG - CGAGTCTCTCTGCGAATCCT |
| Egln3 | TGTCTGGTACTTCGATGCTGA - GCAAGAGCAGATTCAGTTTTTCT |
| Vegfa | TTACTGCTGTACCTCCACC - ACAGGACGGCTTGAAGATG |
| Aqp4 | TGGAGGATTGGGAGTCACC - TGAACACCAACTGGAAAGTGA |
| Tgfb3 | CATGATGATTCCCCCACAC - GCAGTTCTCCTCCAAGTTGC |
| Tnf | TGCCTATGTCTCAGCCTCTTC - GAGGCCATTTGGGAACTTCT |
| Il6 | GCTACCAAACTGGATATAATCAGGA - CCAGGTAGCTATGGTACTCCAGAA |
| Rat |  |
| Hprt | GGTCCATTCCTATGACTGTAGATTTT - CAATCAAGACGTTCTTTCCAGTT |
| Rplp0 | GATGCCCAGGGAAGACAG - ACAATGAAGCATTTTGGATAATCA |
| Cldn5 | GGTCTTTAGCCATGGGGTCT - CAGCCTACCAGACACAGCAC |
| Ocln | ATCTAGAGCCTGGAGCAACG - GTCAAGGCTCCCAAGACAAG |
| Pecam1 | CTCAGTCGGCTGACAAGATG - AGGCTTGCATAGAGCAGCAT |
| Tjp1 | GCATGTAGACCCAGCAAAGG - GGTTTTGTCTCATCATTTCCTCA |

Additional supplementary references

**38.** Drab M, Verkade P, Elger M, et al. Loss of caveolae, vascular dysfunction, and pulmonary defects in caveolin-1 gene-disrupted mice. *Science.* 2001; 293(5539):2449-2452.

**39.** Helms HC, Abbott NJ, Burek M, et al. In vitro models of the blood-brain barrier: An overview of commonly used brain endothelial cell culture models and guidelines for their use. *J Cereb Blood Flow Metab.* 2016; 36(5):862-890.

**40.** Cha S, Johnson G, Wadghiri YZ, et al. Dynamic, contrast-enhanced perfusion MRI in mouse gliomas: correlation with histopathology. *Magn Reson Med.* 2003; 49(5):848-855.

**41.** Ohno-Iwashita Y, Shimada Y, Waheed AA, et al. Perfringolysin O, a cholesterol-binding cytolysin, as a probe for lipid rafts. *Anaerobe.* 2004; 10(2):125-134.

**42.** Gustafsson N, Culley S, Ashdown G, Owen DM, Pereira PM, Henriques R. Fast live-cell conventional fluorophore nanoscopy with ImageJ through super-resolution radial fluctuations. *Nat Commun.* 2016; 7:12471.

**43.** Wegner W, Ilgen P, Gregor C, et al. In vivo mouse and live cell STED microscopy of neuronal actin plasticity using far-red emitting fluorescent proteins. *Sci Rep.* 2017; 7(1):11781.

**44.** Culley S, Albrecht D, Jacobs C, et al. Quantitative mapping and minimization of super-resolution optical imaging artifacts. *Nat Methods.* 2018; 15(4):263-266.

**45.** Schindelin J, Arganda-Carreras I, Frise E, et al. Fiji: an open-source platform for biological-image analysis. *Nat Methods.* 2012; 9(7):676-682.

**46.** Nakagawa H, Fujita T, Izumoto S, et al. cis-diamminedichloroplatinum (CDDP) therapy for brain metastasis of lung cancer. I. Distribution within the central nervous system after intravenous and intracarotid infusion. *J Neurooncol.* 1993; 16(1):61-67.

**47.** Niehoff AC, Grunebaum J, Moosmann A, et al. Quantitative bioimaging of platinum group elements in tumor spheroids. *Anal Chim Acta.* 2016; 938:106-113.
